# Transcriptome Analysis Suggests Dietary Tributyrin Enhances Feeding Intensity via Modulating Steroid Biosynthesis in Mandarin Fish (*Siniperca chuatsi*)

**DOI:** 10.3390/genes16121395

**Published:** 2025-11-21

**Authors:** Er-Xue Xu, Hao-Yu Li, Zhi-Guang Hou, Yi-Huan Xu, Jun Wu, Teng-Fei Bao, Cheng-Bin Wu, Xiao-Wei Gao, Yan-Miao Tan

**Affiliations:** 1 Department of Biology and Health Sciences, Chizhou Vocational and Technical College, Chizhou 247000, China; czyxex@sohu.com (E.-X.X.); maggie7697@126.com (J.W.);; 2 Ocean College, Hebei Agricultural University, Qinhuangdao 066003, China; hyli323@163.com (H.-Y.L.); hzg66888@sina.com (Z.-G.H.); xuyihuan@hebau.edu.cn (Y.-H.X.);; 3 Hebei Key Laboratory of Aquaculture Nutritional Regulation and Disease Control, Qinhuangdao 066003, China

**Keywords:** transcriptome analysis, dietary tributyrin, feeding intensity, steroid biosynthesis, *Siniperca chuatsi*

## Abstract

Blackground/Objectives: Tributyrin (TB), a stable derivative of butyric acid, has been widely used in animal feeds for its health-promoting effects. This study evaluated the efficacy of dietary TB serving as a functional feed additive in enhancing intestinal health of mandarin fish (*Siniperca chuatsi*), with a focus on elucidating the associated molecular mechanisms. Methods: A total of 300 juvenile mandarin fish (200.0 ± 5.2 g) were randomly assigned to one of three dietary groups: a control group (0 mg/kg TB), a low-dose TB group (TB1, 500 mg/kg), or a high-dose TB group (TB2, 1000 mg/kg). The one-month feeding trial was conducted under strictly controlled conditions, with water quality maintained within optimal range. Fish were fed their respective diets twice daily to apparent satiety. Results: Results showed that TB supplementation significantly increased villus height in the mid- and hindgut, with the TB2 group showing the most pronounced improvement. Furthermore, transcriptome analysis revealed that TB altered the expression of genes involved in energy metabolism, fatty acid oxidation, and steroid biosynthesis pathways. Notably, TB supplementation up-regulated key genes such as *gls2b* (energy metabolism) and *cpt1b* (fatty acid oxidation), and modulated especially modulated steroid biosynthesis through genes *sqlea* and *dhcr24h*. Co-expression network analysis further identified hub genes associated with energy metabolism (*etfb*), immune regulation (*il20ra*, *foxp1b*), and cell cycle regulation (*cdc20*, *ccnb1*). Conclusions: These findings elucidate the mechanism of action of TB as a functional feed additive, providing a theoretical foundation for its application in aquaculture to enhance intestinal health.

## 1. Introduction

Studieson animal domestication have been conducted for several years [1]. The domestication of fish with high commercial value can reduce production costs and promote the development of aquaculture. To date, several fish species, including some carnivorous fish, have been successfully domesticated [2,3]. Given that many behavioral traits in fish have a genetic basis, they can respond to artificial selection during domestication. Furthermore, different rearing conditions can lead to growth differences [2]. A major constraint in the culture of the mandarin fish (*Siniperca chuatsi*), an economically significant species in China, is its strong feeding preference for live prey and consequent poor acceptance of formulated diets [4,5]. This feeding habit necessitates the use of live bait, which increases production costs and elevates the risk of disease transmission, thereby limiting the sustainable development of mandarin fish farming. In recent years, considerable research efforts have been directed toward inducing dietary adaptation. For instance, dietary inclusion of 2% soy lecithin has been shown to enhance the growth performance of juvenile mandarin fish [6]. Similarly, supplementation of culture water with Bacillus cereus (10^8^ PFU/mL) improved feed intake, growth, and survival under pellet feed conditions, likely mediated by stimulating innate immunity and remodeling the gut flora [7]. Despite this progress, the unique carnivorous nature of mandarin fish means that artificial compounded feeds often suffer from inherent deficiencies of poor palatability and low nutrient absorption. Therefore, screening for useful additives is necessary to improve the utilization of artificial diets for this species.

Tributyrin (TB), an organic acid ester, is a stable compound composed of three molecules of butyrate and one molecule of glycerol. Its widespread application in animal feeds stems from its efficacy in promoting health and enhancing growth metrics [8,9,10]. TB presented significant advantages as a feed additive due to its mild fatty odor, which improves palatability, and its high stability during processing and storage [11,12]. It is suitable for both monogastric and polygastric species. Upon ingestion, pancreatic lipase hydrolyzes TB in the intestinal lumen, liberating butyric acid and glycerol [12,13]. This targeted release allows more butyrate molecules to exert their functions in the intestine, making TB an effective vehicle for butyrate delivery. TB supplementation improves productivity and health in farm animals by enhancing nutrient absorption and protein digestibility, achieved through its positive impacts on intestinal mucosal structure, barrier integrity, and key physiological processes such as antioxidant defense, inflammatory regulation, and mitochondrial efficiency. Studies have demonstrated the efficacy of TB in promoting growth and preserving intestinal morphology in terrestrial farm animals, including piglets [14,15], small-tailed goats [16], and broilers [17]. In contrast, research on TB in aquatic species remains limited. Existing studies suggest that TB supplementation preserves intestinal health by improving villus integrity, increasing digestive enzyme activities, elevating antioxidant capacity, and suppressing inflammatory responses [18]. For example, in grass carp fed high-soybean meal diets, TB enhanced growth performance and attenuated pro-inflammatory responses by improving intestinal morphology, structural integrity, and microbial composition [19]. However, the application of TB in mandarin fish has been scarcely explored. We speculate that butyrate derived from TB may serve as a direct energy source for the intestinal epithelial cells, thereby promoting villus development, increasing the absorptive surface area, and ultimately improving intestinal health.

Transcriptome profiling is a powerful tool for the global assessment of gene expression, enabling the delineation of biological regulatory networks and their operative mechanisms. It generates comprehensive datasets that facilitate comparative analysis of transcriptional differences across various biological conditions, thereby elucidating gene networks associated with specific traits of interest [20]. In aquaculture research, transcriptomic approaches have become a pivotal method for investigating the genetic underpinnings of key physiological processes such as growth, development, and metabolic functions [21,22]. In the present study, alongside histological analysis, we employed transcriptome sequencing to perform an in-depth analysis of the intestinal tract of mandarin fish fed with TB-supplemented diets. Our objectives were to elucidate TB-mediated regulatory network of intestinal gene expression, delineate its modulatory effects on gut health and physiological function, and establish a scientific foundation for understanding TB’s mechanism of action in fish.

## 2. Material and Methods

### 2.1. Ethical Approval

All experimental procedures were approved by the Animal Experimentation Ethics Committee of Hebei Agricultural University (Approval No. 2023036). All procedures strictly adhered to the standards outlined in the Chinese Laboratory Animal Welfare Guidelines (GB/T 35892-2018 [23]).

### 2.2. Experimental Procedures

A total of 300 uniform-sized juvenile mandarin fish (average body weight of 200.0 ± 5.2 g) were selected from a single cohort sourced from Chizhou, Anhui Province for a one-month feeding trial. The fish were randomly allocated to three experimental groups based on dietary TB supplementation levels: a control group (0 mg/kg TB), a low-dose TB group (500 mg/kg TB), and a high-dose TB group (1000 mg/kg TB), with 100 fish per group (*n* = 100). The fish were reared in separate tanks and fed following a satiety feeding protocol. They were fed twice daily (08:00 and 16:00) until satiety was reached. Detailed information on the formulation and nutritional composition of the mandarin fish diets is provided in Table 1. Water quality parameters were maintained within optimal ranges throughout the trial: temperature, 27 ± 2.0 °C; pH, 7.0 ± 0.5; ammonia nitrogen, <0.15 ± 0.05 mg/L; and dissolved oxygen >5.0 ± 0.5 mg/L.

The experimental period concluded with intestinal tissue sampling, which was performed 12 h after the final feeding. Five fish were randomly sampled from each tank (*n* = 5) for intestinal dissection. The intestines were dissected and divided into three anatomically distinct regions (foregut, midgut, and hindgut). Segments from each region were then processed for different analyses. Samples for histological analysis were fixed in Bouin’s solution at 4 °C, while samples for transcriptomic sequencing were snap-frozen in liquid nitrogen and stored at −80 °C.

### 2.3. Histological Analysis

For histological observation, tissue specimens were dehydrated through a graded ethanol series, cleared in xylene, and embedded in paraffin. Sections (5 μm) were prepared from the embedded blocks and stained with hematoxylin and eosin (H&E). The muscular thickness (MT) and villus height (VH) of the foregut, midgut, and hindgut were measured from the captured images using ImageJ software (version 1.52a).

### 2.4. RNA Extraction, cDNA Library Construction, and RNA-Seq

Total RNA was extracted from samples (five biological replicates per group) using TRIzol reagent (Invitrogen, Carlsbad, CA, USA) according to the manufacturer’s instructions. RNA quality was assessed using an Agilent 2100 Bioanalyzer (Agilent Technologies, Santa Clara, CA, USA). Sequencing libraries were constructed from qualified RNA samples through mRNA enrichment with Oligo (dT) beads, fragmentation, and cDNA synthesis using an Invitrogen kit with random primers. The libraries were prepared through end-repair, poly (A)-tailing, adapter ligation, size selection (200–300 bp) via agarose gel electrophoresis, and PCR amplification. The final libraries were sequenced on an Illumina HiSeq 4000 platform.

### 2.5. Screening of DEGs

Gene expression levels were normalized using the FPKM metric [24]. Differentially expressed genes (DEGs) were identified from the five replicates using the DESeq2 package (version 1.20.0) [25], which employs a negative binomial generalized linear model to account for read count variability. The thresholds for DEG screening were set at |log_2_ fold change| ≥ 1 and *p*-adj < 0.05. To functionally characterize the identified DEGs, enrichment analyses of Gene Ontology (GO) terms and Kyoto Encyclopedia of Genes and Genomes (KEGG) pathways were performed. Significantly enriched terms were detected by comparing the DEG set against the genomic background using a hypergeometric test.

### 2.6. Co-Expression Network Construction and Module Identification

A weighted gene co-expression network analysis (WGCNA) was performed using the WGCNA R package (version 1.47) to identify co-expressed gene modules [26]. The optimal soft-thresholding power for network [27] construction was determined to be 30, achieving a scale-free topology fit index (R^2^) of 0.85. Co-expression networks were subsequently generated with parameters minModuleSize = 30 and mergeCutHeight = 0.25. Module eigengene-based correlation coefficients were calculated to identify biologically relevant modules. Intramodular connectivity (functional soft connectivity) was computed for each gene, and genes ranking in the top 1% or 5% for connectivity within their respective modules were designated as hub genes. The resulting network was visualized using Cytoscape software package (v3.3.0).

### 2.7. Construction and Analysis of Bayesian Networks

A gene regulatory network (GRN) was constructed using the CBNplot R package (version 1.2.1), with genes from Weighted Gene Co-Expression Network Analysis (WGCNA) modules that correlated with TB supplementation. Genes significantly associated with TB supplementation (FDR < 0.05), identified based on a previously reported Bayesian model [28,29], were used to build the Bayesian network.

### 2.8. Confirmation of RNA-Seq Data by qRT-PCR

To validate the RNA-seq data, eight selected DEGs were analyzed by quantitative real-time PCR (qRT-PCR). Total RNA was isolated from the intestines of mandarin fish in C, TB1 andTB2 groups. Gene-specific primers were designed and synthesized by Sangon Biotech (Shanghai, China; Table S1). First-strand cDNA was synthesized from total RNA using the Hiscript III Reverse Transcriptase kit (Vazyme, Nanjing, China) according to the manufacturer’s protocol. qPCR reactions were performed in a 10 μL volume containing 5.0 μL of 2× ChamQ Universal SYBR qPCR Master Mix (Vazyme), 1 μL of cDNA template, 0.5 μL of each forward and reverse primer (10 μM; Table 1), and 3 μL of nuclease-free water. The thermal cycling conditions were as follows: initial denaturation at 95 °C for 30 s; 40 cycles of 95 °C for 5 s and 60 °C for 30 s; followed by a melting curve analysis of 95 °C for 15 s, 60 °C for 1 min, and 95 °C for 15 s [30]. Each sample was run in three technical replicates, and β-actin was used as the reference gene for normalization. The relative expression of target genes was calculated using the 2^−ΔΔCt^ method [31].

### 2.9. Statistical Analysis

The significance of intergroup differences for all measured parameters was determined by one-way analysis of variance (ANOVA) using SPSS (v26; IBM Corp., Armonk, NY, USA). When ANOVA indicated a significant effect, Tukey’s HSD test was applied for post hoc comparisons. Differences were considered statistically significant at *p* < 0.05.

## 3. Results

### 3.1. Histological Comparison

Histological analysis revealed significant variations in intestinal villus height (VH) and muscular thickness (MT) across different gut segments and dietary treatments (Figure S1). In the foregut, no significant differences in VH were observed among the groups. However, the MT in the control group (C) was significantly greater than that in both the TB1 and TB2 groups. In the midgut, VH was significantly increased in both the TB1 and TB2 groups compared to the C group, with no significant difference between the two treatment groups. Regarding MT, no significant difference was found between the C and TB1 groups, but both exhibited greater thickness than the TB2 group. In the hindgut, VH exhibited a dose-dependent increase, with the TB2 group showing the highest value, followed by TB1, and then the C group. Conversely, MT was significantly greater in both TB1 and TB2 groups compared to the C group, with no significant difference between the two treatment groups (Figure S2).

### 3.2. Intestinal RNA-Seq Analysis

To investigate molecular-level alterations induced by TB supplementation, we conducted transcriptomic analysis on the intestinal tract of mandarin fish fed diets containing 0, 500, or 1000 mg/kg TB. The raw sequencing data from all 15 samples have been deposited in the NCBI database. Sequencing generated 668,511,348 raw reads, from which 658,845,572 high-quality clean reads were obtained after quality control. All key quality metrics, including Q20 (97.84–98.35%), Q30 (93.89–95.39%), and GC content (46.34–47.38%), fell within acceptable ranges, confirming the data’s suitability for subsequent analyses.

**Figure 1:**
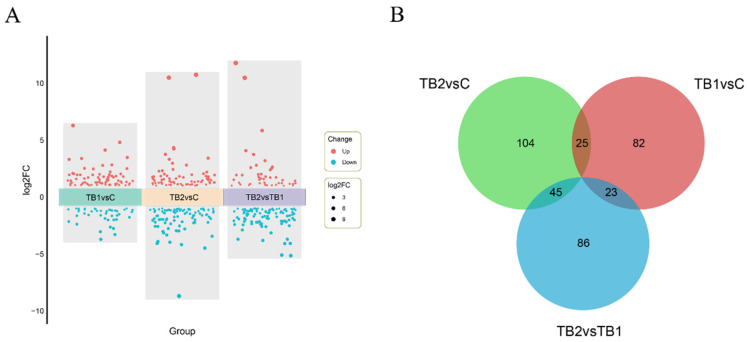
Transcriptomic profiling of intestinal tissue from mandarin fish after tributyrin (TB) supplementation. (**A**) Volcano plot illustrating differentially expressed genes (DEGs) between TB-treated and control groups. The x-axis denotes experimental groups, while the y-axis indicates the log2 fold change (log2FC). Each data point represents an individual gene, with significantly differentially expressed genes (|log_2_FC| ≥ 1 & *p*-adj < 0.05) highlighted. Point size corresponds to the absolute value of log2 fold change. Significantly up-regulated DEGs are colored orange, while down-regulated DEGs are in cyan. (**B**) Venn diagram depicting the overlap of DEG sets across experimental comparisons. Numbers in overlapping and non-overlapping regions indicate the count of unique and common DEGs for each comparison, respectively.

Differential expression analysis identified 121 DEGs between TB1 and C, 164 between TB2 and C, and 144 between TB2 and TB1 (Figure 1A). A Venn diagram illustrated the overlap among these comparisons, showing 25 DEGs common to both TB1 vs. C and TB2 vs. C, 23 shared between TB1 vs. C and TB2 vs. TB1, and 45 overlapping between TB2 vs. C and TB2 vs. TB1 (Figure 1B).

**Figure 2:**
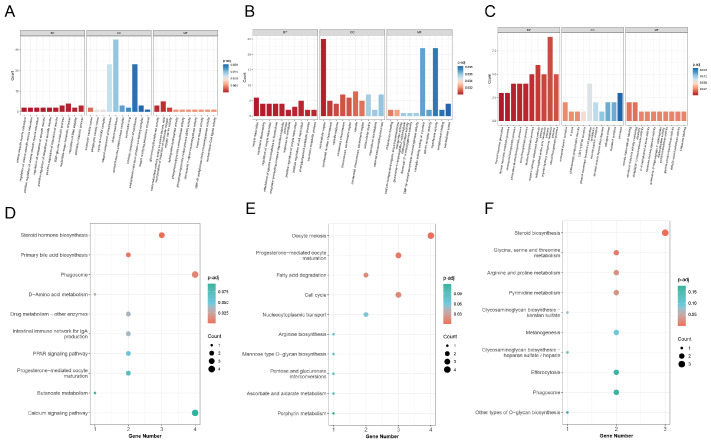
GO terminology and DEG enrichment in the KEGG pathway. (**A**–**C**) The ten most significantly enriched Gene Ontology terms for the pairwise comparisons TB1 vs. C, TB2 vs. C, and TB2 vs. TB1. (**D**–**F**) The top ten enriched KEGG pathways for the respective comparisons; the size of the data points corresponds to the number of DEGs assigned to each pathway.

The functional characterization of the DEGs was performed through Gene Ontology (GO) enrichment analysis across the three standard categories: biological process (BP), cellular component (CC), and molecular function (MF). The ten most significantly enriched GO terms for each comparison are displayed in Figure 2A–C. In the TB1 vs. C comparison, the most significantly enriched GO terms were primarily associated with the plasma membrane and its intrinsic and integral components. For TB2 vs. C, DEGs were mainly enriched in the extracellular region, catalytic activity acting on proteins, and transferase activity. The TB2 vs. TB1 comparison showed significant enrichment in metabolic and biosynthetic processes, including organic hydroxy compound metabolism, alcohol biosynthesis, and cellular modified amino acid metabolism.

KEGG pathway enrichment analysis was also performed, with scatter plots visualizing the ten most significantly enriched pathways for each comparison (Figure 2D–F). The most significantly enriched pathways in the TB1 vs. C comparison were Steroid hormone biosynthesis, Phagosome, and Primary bile acid biosynthesis. For TB2 vs. C, enrichment was observed in 80 pathways, notably including Oocyte meiosis, Progesterone-mediated oocyte maturation, Fatty acid degradation, and Cell cycle. The TB2 vs. TB1 comparison revealed primary enrichment in Steroid biosynthesis, Glycine, serine and threonine metabolism, Arginine and proline metabolism, and Pyrimidine metabolism pathways. These results indicate that TB supplementation substantially influences steroid and lipid biosynthesis, as well as broader metabolic processes, in the intestinal tissues of mandarin fish.

### 3.3. WGCNA and Bayesian Analysis

**Figure 3:**
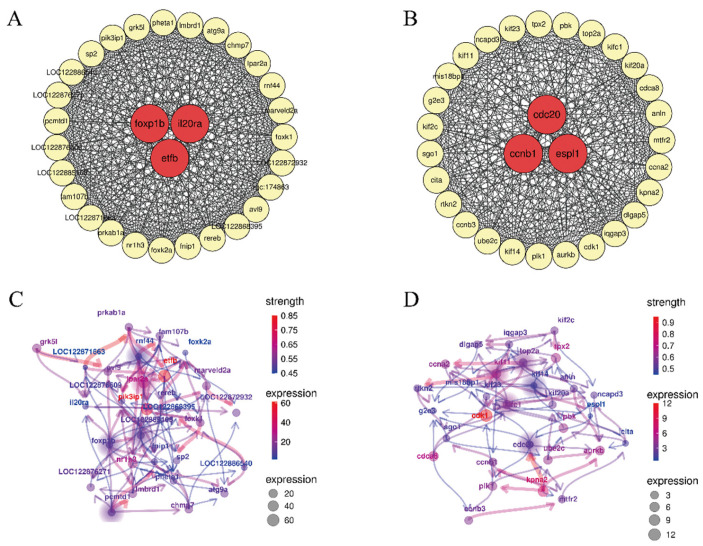
Co-expression network of 30 genes in MEturquosie (**A**) and MEmagenta (**B**). Representation of key genes predicted by Bayesian network analysis in the MEturquoise (**C**) and MEmagenta (**D**) modules. Key genes are marked by solid circles. The color gradient of the edges (blue to red) indicates the strength of the inferred interactions.

WGCNA identified two functional modules that exhibited significant correlations with TB supplementation. The expression profile of the MEturquoise module was significantly correlated with the TB1 group, while the MEmagenta module was strongly associated with the TB2 group (Figure S3). Hub gene analysis within each module revealed three primary hub genes in the MEturquoise module (*foxp1b*, *il20ra*, and *etfb*; containing 30 genes in total) and three in the MEmagenta module (*cdc20*, *ccnb1*, and *espl1*; Figure 3A,B). Subsequently, a set of thirty pivotal genes from the MEturquoise and MEmagenta modules was used to construct a Bayesian network model of differentially expressed genes (DEGs) using the CBNplot R package (https://github.com/noriakis/CBNplot, accessed on 20 November 2024) (Figure 3C,D). Within the MEturquoise module, the Bayesian model identified *rnf44*, *lpar2a*, *foxp1b*, and *pcmtd1* as key regulatory genes. Similarly, *top2a*, *kif11*, *cdc20*, and *kif14* were identified as key genes within the MEmagenta module.

### 3.4. qRT-PCR Validation of Gene Expression

**Figure 4:**
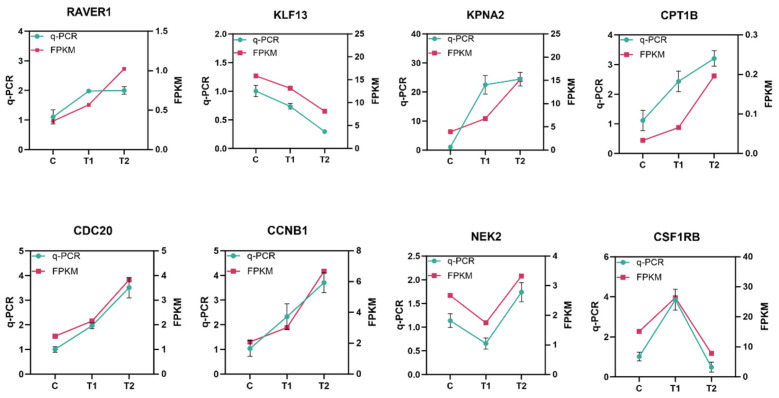
Confirmation of RNA-Seq results through qRT-PCR quantification of eight randomly selected DEGs.

The expression levels of eight selected genes (*raver1*, *klf13*, *kpna2*, *cpt1b*, *cdc20*, *ccnb1*, *nek2*, and *csf1rb*) were validated by qRT-PCR and compared with the RNA-Seq results (FPKM values). As illustrated in Figure 4, the expression patterns (up- or down-regulation) of all eight genes determined by qRT-PCR were consistent with the trends observed in the transcriptome data. The close agreement between the two methods confirms the reliability of our transcriptomic analysis.

## 4. Discussion

The intestinal tract is critical to fish physiology, performing essential functions in digestion and nutrient absorption. Villus height and muscular thickness are key morphological parameters indicative of its functional integrity and health status [32,33]. Segment-specific responses in these parameters are vital for mandarin fish to adapt to artificial feed. Previous research indicates that intestinal villus shortening, often associated with inflammatory conditions [34], reduces absorption efficiency by lessening digesta-epithelium contact [35], potentially explaining mandarin fish’s poor artificial feed. Additionally, the shortening of midgut and hindgut villus in the control group was more pronounced than that reported in a previous study [36]. In the present study, TB modulated mandarin fish intestinal morphology under artificial feeding, increasing midgut and hindgut villus height and altering muscular thickness across segments such as lower foregut muscular thickness in TB groups. These effects align with findings in grass carp [7] and yellow catfish [37], indicating TB’s cross-species role in enhancing intestinal morphology to facilitate nutrient absorption and its potential to improve artificial feed utilization in mandarin fish. Yet, TB inhibited foregut muscular thickness in mandarin fish, likely due to the foregut’s role in physical digestion where TB may adjust smooth muscle contraction to align with midgut and hindgut absorption. Additionally, midgut/hindgut villus shortening in the control group was more pronounced than that in a previous study [38], possibly attributable to variations in feed composition, genetics, or aquaculture conditions.

As gene expression regulates complex physiological processes, we employed transcriptome sequencing was to analyze the molecular effects of TB-supplemented artificial feed on intestine of mandarin fish, aiming to elucidate the TB’s mechanism of action. Key pathways exhibited significant enrichment of DEGs, including Steroid hormone biosynthesis, Cell cycle, and Fatty acid metabolism. In the TB1 vs. C group, *gls2b* within the D-amino acid metabolism pathway was significantly upregulated. Although the function of this gene in mandarin fish has not been directly validated, human *gls2* can catalyze the conversion of glutamine to glutamate, thereby participating in energy metabolism and oxidative stress regulation [39]. Given that intestinal epithelial cells in mandarin fish rely on glutamate for energy supply and require it to maintain redox balance [40], it is likely that the mandarin fish *gls2b* gene exhibits functional conservation. By upregulating this gene, TB may supply energy for the proliferation and repair of intestinal epithelial cells and boost the intestinal antioxidant capacity. This finding is consistent with the morphological observation of increased midgut villus height in the TB1 group, offering molecular-level evidence for TB’s role in enhancing intestinal morphology. In the TB2 and C comparison, the *ccnb1*, *cdc20*, and *plk1* genes within the cell cycle pathway were significantly upregulated. *Ccnb1* facilitates the G2/M phase transition of the cell cycle [41], while *plk1* is a critical regulator of the cell cycle in vertebrates [42]. The upregulation of these genes correlates with the highest hindgut villus height observed in the TB2 group. This suggests that TB promotes intestinal cell proliferation in mandarin fish by activating cell cycle-related genes, consistent with studies in other fish showing the involvement of cell cycle genes in intestinal development regulation [43]. Meanwhile, the *cpt1b* and *gcdha* genes within the fatty acid metabolism pathway were also significantly upregulated. Although short-chain fatty acids derived from TB hydrolysis do not require *cpt1b* for transport, and *cpt1b* is predominantly expressed in muscle tissues in fish [44], its upregulation in mandarin fish intestine suggests that TB may enhance mitochondrial function to boost metabolic activity, thereby providing energy for proliferating intestinal cells. Given the limited research on the *gcdha* gene in fish, its potential auxiliary role in intestinal fatty acid metabolism can only be inferred from reports of its homologous genes participating in fatty acid β-oxidation [45]. Further experimental validation is required to determine its specific function. In the TB2 vs. TB1 comparison, *sqlea*, *msmo1*, and *dhcr24h* within the steroid synthesis pathway were significantly upregulated. Previous studies have shown that under stress conditions, such as exposure to trivalent chromium in coral trout [46] and low-temperature treatment in grass carp [47], the steroid synthesis pathway is activated to enhance lipid metabolism. The TB-induced activation of this pathway in the present study appears consistent with this core mechanism, suggesting that high-concentration TB may promote the conversion of lipids from artificial feed into cholesterol. Notably, the activation of this pathway was concentration-dependent, potentially representing a unique molecular regulatory strategy employed by mandarin fish to adapt to artificial feed. Furthermore, this finding may explain the superior gut health observed in the TB2 group—likely achieved through the synergistic action of multiple pathways, including cell proliferation, energy metabolism, and steroid synthesis, thereby comprehensively regulating intestinal function.

While previous research has established that TB supplementation enhances intestinal health in fish, the complex co-expression networks modulated by TB remain relatively unexplored. Unsurprisingly, our WGCNA of mandarin fish transcriptome data revealed that two modules, designated turquoise and magenta, showed a strong correlation with TB supplementation. Genes within both modules were enriched in energy metabolism, immune regulation, and cell cycle pathways, indicating these biological processes play pivotal roles in mediating responses to TB-containing diets. Several central hub genes were identified in both modules. In the turquoise module, for example, *il20ra* was identified as a hub gene. In mammals, IL-20RA signaling can disrupt the epithelial barrier by reducing filaggrin expression [48]. Although its function in fish requires validation, this suggests a potential conserved role in modulating mucosal integrity. *Foxp1b* is a transcription factor associated with Treg cell differentiation and suppression of excessive inflammation in mammals [49]. *Foxp1b* is likely to play a similar role in fish, promoting Treg-like cells to suppress intestinal inflammation and aid in tissue repair. Electron transport flavoprotein subunit β (*Etfb*) is essential for mitochondrial fatty acid oxidation, which provides energy for cellular processes [50]. In the magenta module, three mitosis-related hub genes were identified. *cdc20* and *ccnb1* were significantly enriched in the cell cycle pathway. The need for rapid renewal of the fish intestine may be associated with the high expression of *cdc20* and *ccnb1*, and TB may promote rapid repair after intestinal injury by accelerating the cell cycle.

While this study provides valuable insights into the transcriptomic response to TB supplementation, several limitations should be acknowledged. The observed gene expression patterns, although significant, represent associations rather than definitive causal relationships. Additionally, the potential role of gut microbiota as a confounding factor cannot be overlooked, as TB-derived butyrate might mediate its effects through microbiome interactions. Furthermore, the restriction of our analysis to intestinal tissue limits the generalizability of these findings to other metabolic organs. These considerations highlight the importance of future research incorporating mechanistic approaches, microbiome assessment, and multi-tissue analyses to fully understand TB’s physiological effects.

## 5. Conclusions

RNA sequencing demonstrated that dietary TB supplementation upregulates genes involved in energy metabolism (*gls2b*) and fatty acid oxidation (*cpt1b*), thereby enhancing intestinal function through increased energy production and reduced oxidative stress. Furthermore, TB modulated steroid biosynthesis genes (*sqlea*, *msmo1*, *dhcr24h*) in a dose-dependent manner, a process that promotes the conversion of lipids to cholesterol. Co-expression network analysis identified hub genes involved in energy metabolism (*etfb*), immune regulation (*il20ra*, *foxp1b*), and cell cycle control (*cdc20*, *ccnb1*, *plk1*), highlighting the multi-pathway role of TB in maintaining intestinal homeostasis. Morphological assessment confirmed a significant increase in intestinal villus height, with the TB1 group displaying improved midgut villus architecture and the TB2 group achieving the highest villus height in the hindgut. Collectively, these findings elucidate the mechanistic actions of TB as a functional feed additive and indicate its potential applications in aquaculture to enhance growth efficiency and feed conversion rates in fish.

## Figures and Tables

**Table 1:** Nutritional composition of the experimental diets for mandarin fish.

Item	Content (%)
Crude protein	≥50%
Crude fat	≥9%
Crude fiber	≤5%
Coarse ash	≤12%
Lysine	≥3%
Calcium	≥1.8%
Total phosphorus	≥1.8%
Moisture	≤12%

## Data Availability

The original contributions presented in the study are included in the article/Supplementary Materials; further inquiries can be directed to the corresponding author.

## Supplementary Materials

The following supporting information can be downloaded at: https://www.mdpi.com/article/10.3390/genes16121395/s1, Table S1. Specific primers for eight verified genes used for qRT-PCR; Figure S1. Effects of different concentrations of TB on the morphology of foregut (A,D,G), midgut (B,E,H) and hindgut (C,F,I) in groups C, TB1 and TB2. Images were captured by light microscopy after H&E staining (magnification × 4). MT, Intestinal muscular thickness; VH, Intestinal villus height; scale bar = 200 µm; Figure S2. Intestinal muscle thickness (MT), and intestinal villus height (VL) were studied for all intestinal sections. (A-C) Height of intestinal villi in the foregut, midgut, and hindgut. (D–F) Intestinal muscle thickness of anterior, middle, and posterior. All data are shown as the mean ± standard deviation. Significant differences (*p* < 0.05) between groups, as determined by one-way ANOVA, are indicated by different superscript letters; Figure S3. Relationships between modules and TB treatment. The color scale on the right shows module-trait correlation coefficients from −1 (blue) to 1 (red), indicating low to high correlations. All raw data generated in this study have been deposited in the NCBI SRA database (PRJNA1348964).
