# Supplementary material for: Transcriptome Analysis Suggests Dietary Tributyrin Enhances Feeding Intensity via Modulating Steroid Biosynthesis in Mandarin Fish (Siniperca chuatsi)
Source: Genes (Basel). 2025 Nov 21;16(12):1395. doi: 10.3390/genes16121395 (PMC12733369; doi:10.3390/genes16121395)
Supplement: Supplementary file 1 [file genes-16-01395-s001.zip › genes-3974585-supplementary.pdf]

Table S1. Specific primers for eight verified genes used for qRT-PCR.

Gene name	Primer sequences (5′-3′)
raver1	TTTGCGGTGTTGGAGTTTGC
	AATCAGAGCAGCCAACATGC
klf13	AGACTCCCAACAATGTTGCC
	TTCGACCTCGCGATCTTTTG
kpna2	TCAGACAGCTGCTGTTGTTG
	ACCAGCAATGTTTCCAAGGG
cpt1b	TGCCATCATGTTTGCCACTG
	GCCACACTTTTGTTGACGTG
cdc20	ATCACAAATGCTCCCATGGC
	AAACTCTGCGACACATTGCC
ccnb1	GGTGCTCAAGTTCAAACTAGGC
	ATTTTGCCAGGGTGTGTTGC
nek2	ACATTGTCATGGAGCACTGC
	ACGCCTTTCCTTGATGCATC
csf1rb	ACGGCAACAACAACATGGTG
	AGCCGCTGAAGAAGGAGTTAG
β-actin	ATCGCCGCACTGGTTGTTGAC
	CCTGTTGGCTTTGGGGTTC

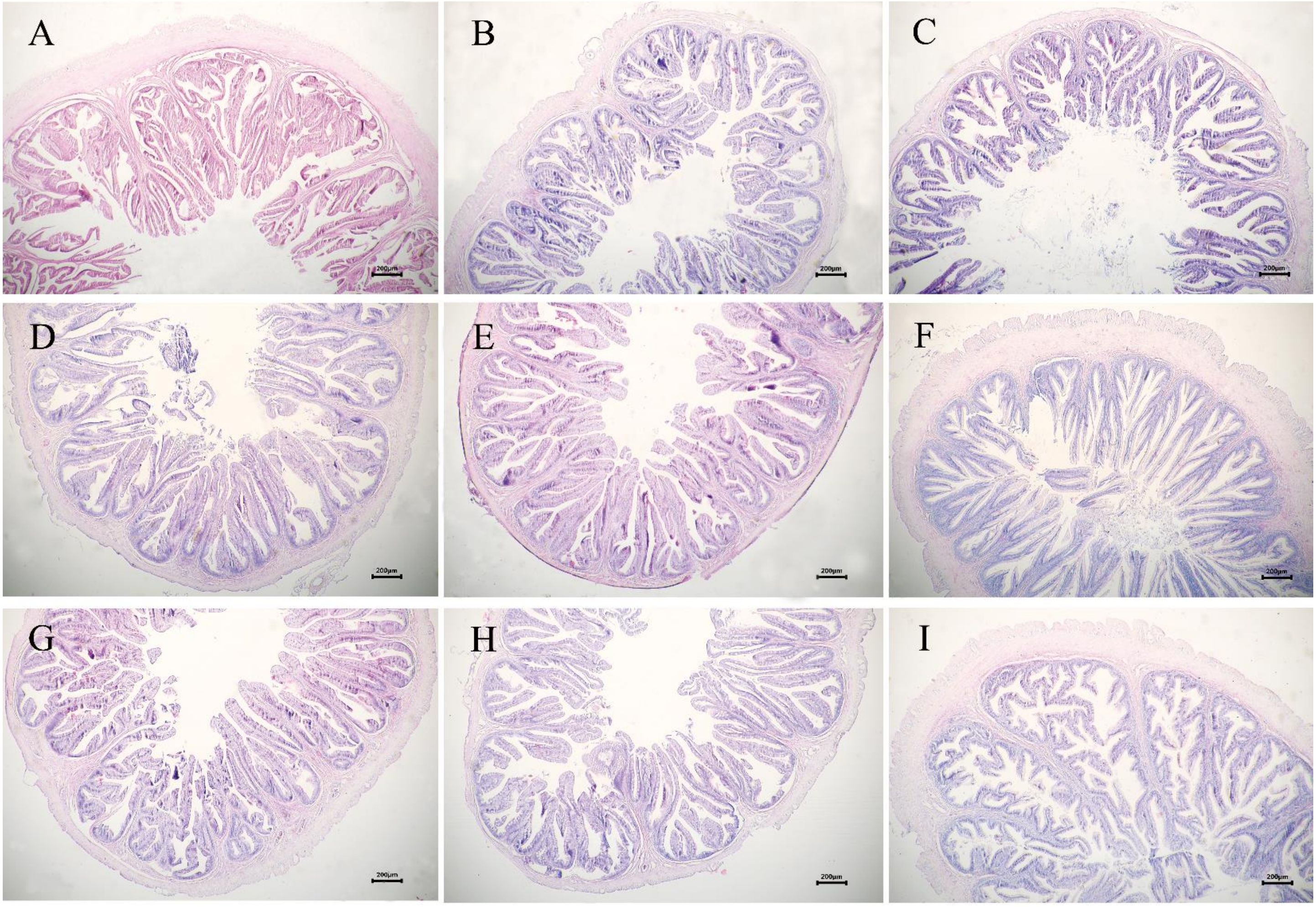

Figure S1. Effects of different concentrations of TB on the morphology of foregut (A, D, G), midgut (B, E, H) and hindgut (C, F, I) in groups C, TB1 and TB2. Images were captured by light microscopy after H&E staining (magnification × 4). MT, Intestinal muscular thickness; VH, Intestinal villus height; scale bar = 200 μm.

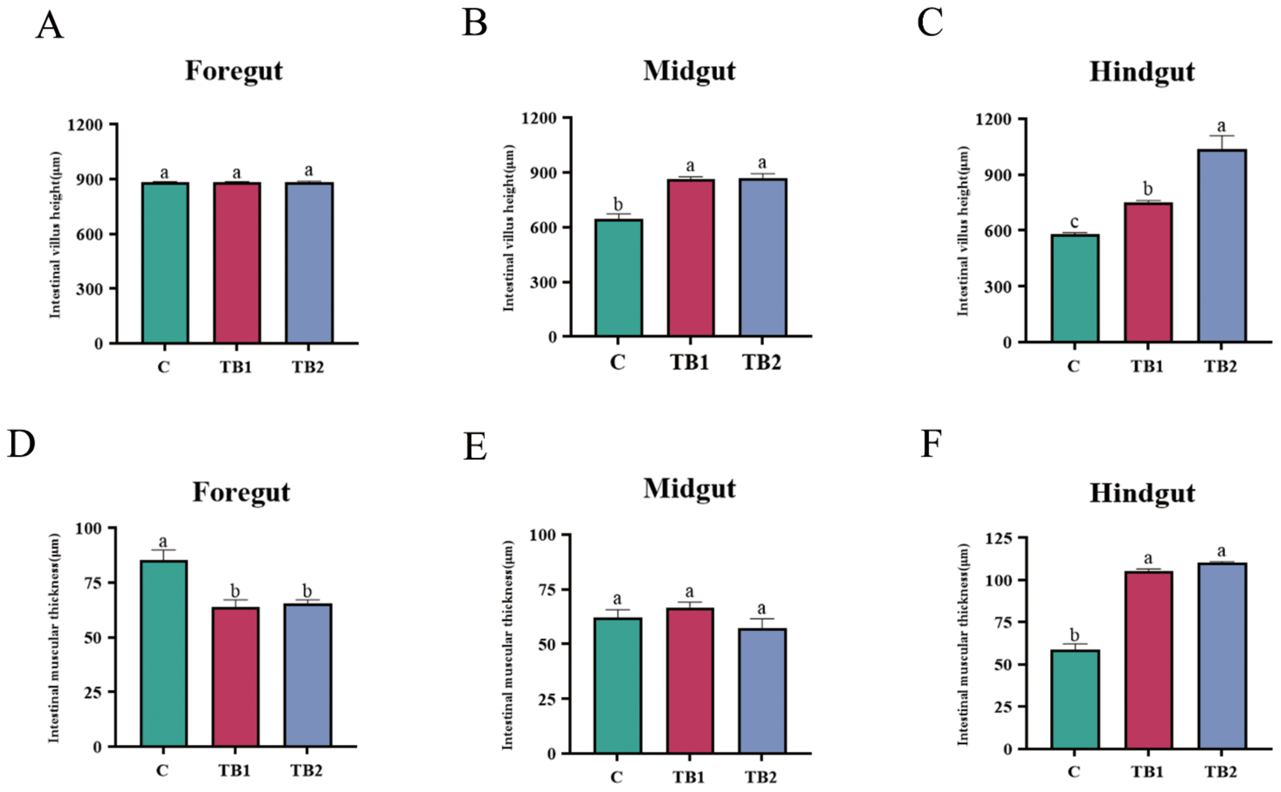

Figure S2. Intestinal muscle thickness (MT), and intestinal villus height (VL) were studied for all intestinal sections. (A-C) Height of intestinal villi in the foregut, midgut, and hindgut. (D-F) Intestinal muscle thickness of anterior, middle, and posterior. All data are shown as the mean ± standard deviation. Significant differences ($p < 0.05$) between groups, as determined by one-way ANOVA, are indicated by different superscript letters.

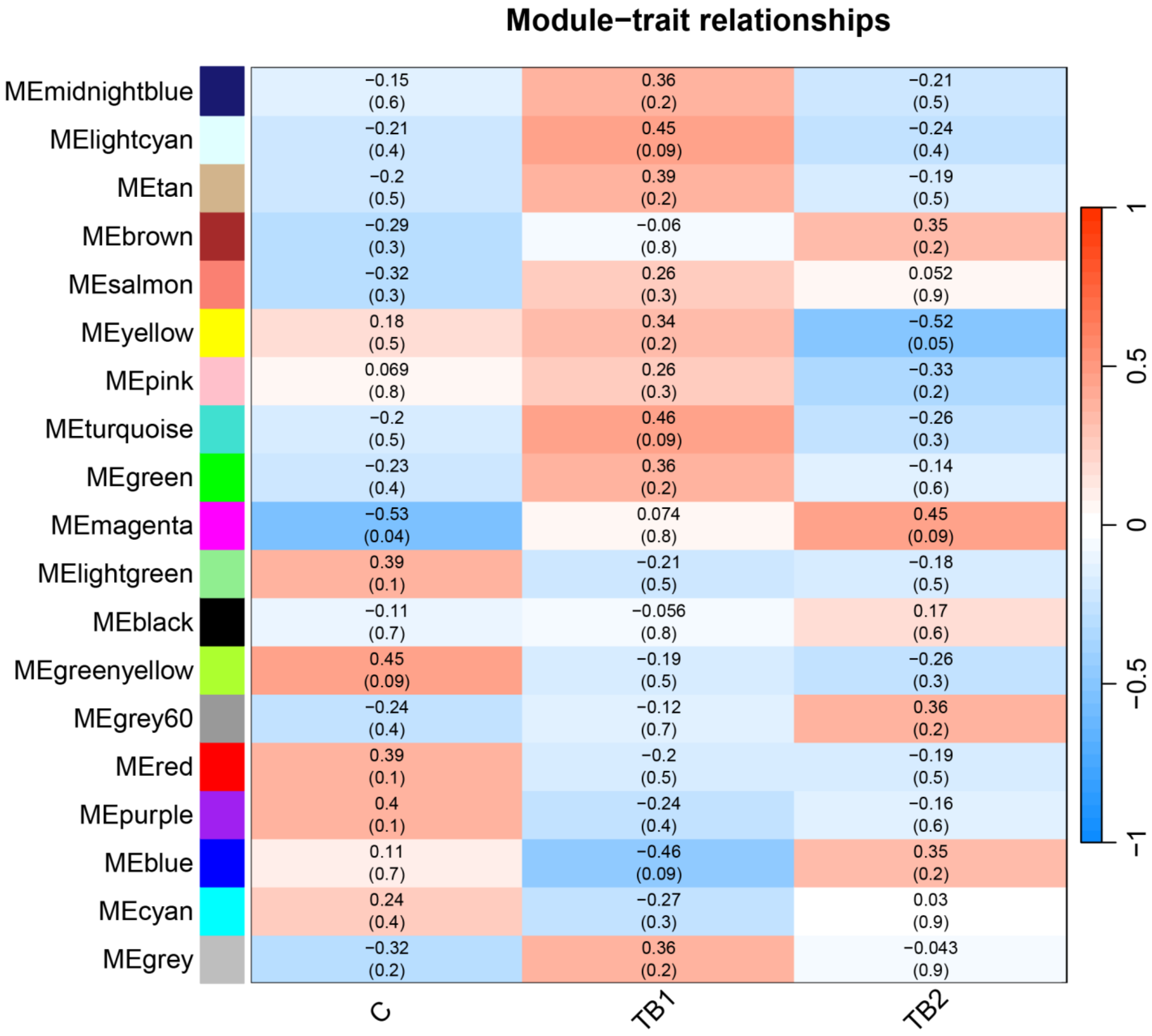

Figure S3. Relationships between modules and TB treatment. The color scale on the right shows module-trait correlation coefficients from −1 (blue) to 1 (red), indicating low to high correlations.
